# Role of RKKY torque on domain wall motion in synthetic antiferromagnetic nanowires with opposite spin Hall angles

**DOI:** 10.1038/s41598-017-11733-9

**Published:** 2017-09-15

**Authors:** S. Krishnia, P. Sethi, W. L. Gan, F. N. Kholid, I. Purnama, M. Ramu, T. S. Herng, J. Ding, W. S. Lew

**Affiliations:** 1 School of Physical and Mathematical Sciences, Nanyang Technological University, 21 Nanyang Link, 637371 Singapore, Singapore; 2 Department of Materials Science and Engineering, National University of Singapore, 9 Engineering Drive 1, 117576 Singapore, Singapore

## Abstract

We experimentally show the effect of enhanced spin-orbit and RKKY induced torques on the current-induced motion of a pair of domain walls (DWs), which are coupled antiferromagnetically in synthetic antiferromagnetic (SAF) nanowires. The torque from the spin Hall effect (SHE) rotates the Néel DWs pair into the transverse direction, which is due to the fact that heavy metals of opposite spin Hall angles are deposited at the top and the bottom ferromagnetic interfaces. The rotation of both DWs in non-collinear fashion largely perturbs the antiferromagnetic coupling, which in turn stimulates an enhanced interlayer RKKY exchange torque that improved the DW velocity. The interplay between the SHE-induced torque and the RKKY exchange torque is validated via micromagnetic simulations. In addition, the DW velocity can be further improved by increasing the RKKY exchange strength.

## Introduction

Recent observations of fast current-driven domain wall (DW) motion under the influence of spin Hall effect (SHE) in ferromagnetic (FM) materials with perpendicular magnetic anisotropy (PMA) have led to the development of novel spintronic devices. For instance, Haazen *et al*. has reported on the current-driven Néel DW motion under the influence of SHE in both electron and current flow directions by tuning the Pt thicknesses in Pt/Co/Pt stacks^1^. Beach *et al*. observed an enhanced SHE in Pt/Co/Ta stack, which revealed the opposite sign of SHE in Ta and Pt^2^. Later, the stabilization of the Néel DWs without any external magnetic field in micron-sized wires was attributed to the Dzyaloshinskii-Moriya Interaction (DMI) which originated from the heavy-metal/ferromagnetic interfaces^3–6^. The DMI locks internal spins texture of a DW into Néel configuration which allows for much faster DW speeds upon driven by current^3–5^. In addition, high speed DW wall motion have also been shown in coupled PMA nanowires and synthetic antiferromagnetic (SAF) PMA nanowires by making use of antiferromagnetically coupled Néel DWs^7,8^. DW motion in SAF nanowires provides a helpful design for DW memory device^8^ as it eliminates the DW stray fields^9–11^. The current-induced DW motion in the SAF nanowires is explained by an additional torque which arises due to RKKY antiferromagnetic coupling^8^.

Here, we study the enhanced current-induced DW motion in a perpendicularly-magnetized SAF nanowire due to the high SHE and RKKY-induced torques. The high SHE is attributed to the presence of heavy metals of opposite spin Hall angle at the top and bottom interfaces. When current is applied, the DWs in the SAF nanowire become non-collinear due to the SHE-induced torques that act on the DWs in same direction at both the top and the bottom interfaces. This in turn stimulates an enhanced RKKY interlayer exchange torque which further improves the DW velocity as compared to the previous study^8^. The interplay between the SHE torque and the RKKY exchange torque is clarified via micromagnetic simulations. Additionally, our calculations and micromagnetic simulations show that the magnitude of the RKKY exchange torque increases with respect to the perturbation in the antiferromagnetic coupling and it is maximum when both the DWs are perpendicular to each other.

## Results and Discussion

**Figure 1:**
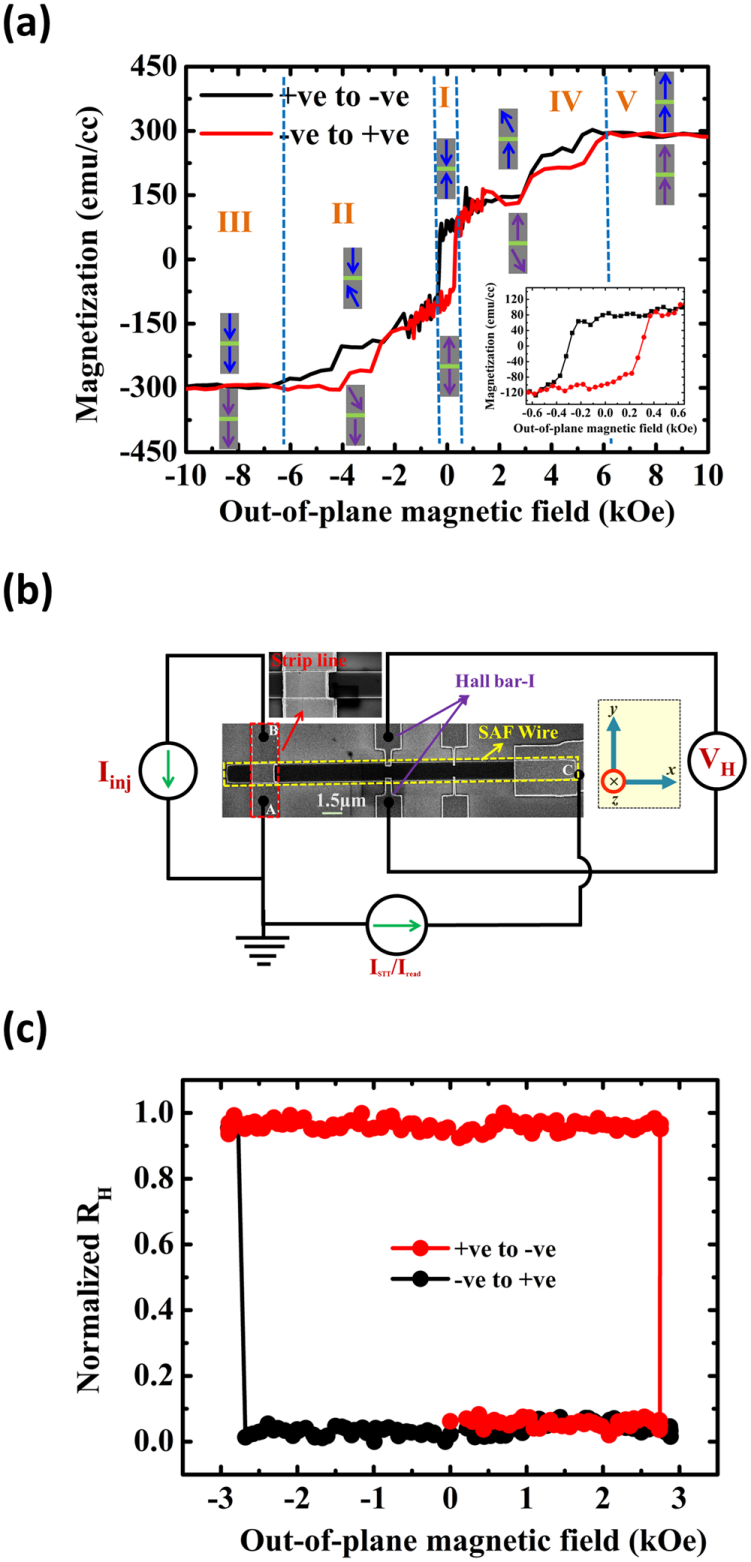
(**a**) Out-of-plane VSM hysteresis loop measurement of the Ta(3)/Pt(3)/[Co(0.4)/Ni(0.7)/Co(0.4)]/Ru(0.8)/[Co(0.4)/Ni(0.7)/Co(0.4)]/Ta(3) SAF stack. The multiple magnetization reversal processes in the measurement indicates the antiferromagnetic coupling between two magnetic trilayer Co(0.4)/Ni(0.7)/Co(0.4) structures. Each region drawn in the hysteresis loop is illustrated with the relevant magnetic configurations of the SAF structure. Inset shows the hysteresis loop close-up of region I. **(b)** Scanning electron microscope (SEM) image with measurement circuit schematic. Inset shows the close-up of the strip line. **(c)** Normalized R-H loop measurement of the device, corresponding to region I, was obtained using anomalous Hall effect measurement technique. During the Hall measurement, an external magnetic field was swept perpendicularly to the plane of the device.

Figure 1(a), shows the measured hysteresis loop of a Ta(3)/Pt(3)/[Co(0.4)/Ni(0.7)/Co(0.4)]/Ru(0.8)/[Co(0.4)/Ni(0.7)/Co(0.4)]/Ta(3) thin film stack. The hysteresis of the thin film exhibits magnetization switching at 380 Oe, whereas reversible nature of the hysteresis in the field range 500 Oe to 6.2 kOe revels that magnetization reversal process is achieved by magnetization rotation in the high field regime. The multiple magnetization reversal processes in the hysteresis can be attributed to the antiferromagnetic coupling between the bottom (M_1_) and the top (M_2_) magnetic Co/Ni/Co trilayers that are separated by an ultrathin Ru spacer layer. At high magnetic fields, both layers are saturated (M = |M_1_ + M_2_| ~300 emu/cc) along the external applied field direction, as shown in regions III and V. Regions II and IV show the flipping of free layer. Here, the top (M_2_) and the bottom (M_1_) layers are assumed as the free and the hard layers, respectively. The region around zero magnetic fields, *i.e*. region I, is where the spins of both the magnetic layers are aligned antiparallel to each other. The spin configurations of both layers in all five regions are represented by the blue and purple arrows. The blue (purple) arrows represent the magnetic configuration of the layers when the system was swept from high (low) field to low (high) field. Inset of Fig. 1(a) shows the enlarged hysteresis of region I. The coercivity of our SAF stack in region I was found to be ~380 Oe with a hard axis anisotropy field *H*_*K*_ > 5.5 kOe. The coercivity of the SAF thin film stack is much higher than our single and double stack PMA of the same material, as shown in Supp. S1. Both the increase in the coercivity and the multiple magnetization reversal process in the hysteresis confirm the presence of RKKY antiferromagnetic (AFM) coupling between the two magnetic layers^12^. The energy associated with the RKKY exchange coupling was calculated as *E*_*ex*_ = *M*_*s*_ *tH*_*RKKY*_ = 0.54 erg/cm^2^, which is as large as values reported in literature^13^. Here *M*_*s*_ is the saturation magnetization, *H*_*RKKY*_ is the interlayer exchange field, and *t* is the thickness of thin film.

To study the DW dynamics in our SAF stack, 30 µm long and 1.5 µm wide nanowires were fabricated using electron beam lithography and Ar ion-milling techniques. Figure 1(b) shows the scanning electron microscopy (SEM) image of the device with the electrical contacts schematic. The normalized Hall resistance (*R*_*H*_) of the nanowire without any prior DW injection is obtained by sweeping an out-of-plane field, and the result is shown in Fig. 1(c). The normalized *R*_*H*_ value of 1 (0) corresponds to the upward (downward) direction of magnetization at the Hall bar. The square hysteresis loop, which corresponds to region I of the thin film stack, with a coercivity of ∼2750 Oe confirms the perpendicular magnetic easy axis.

**Figure 2:**
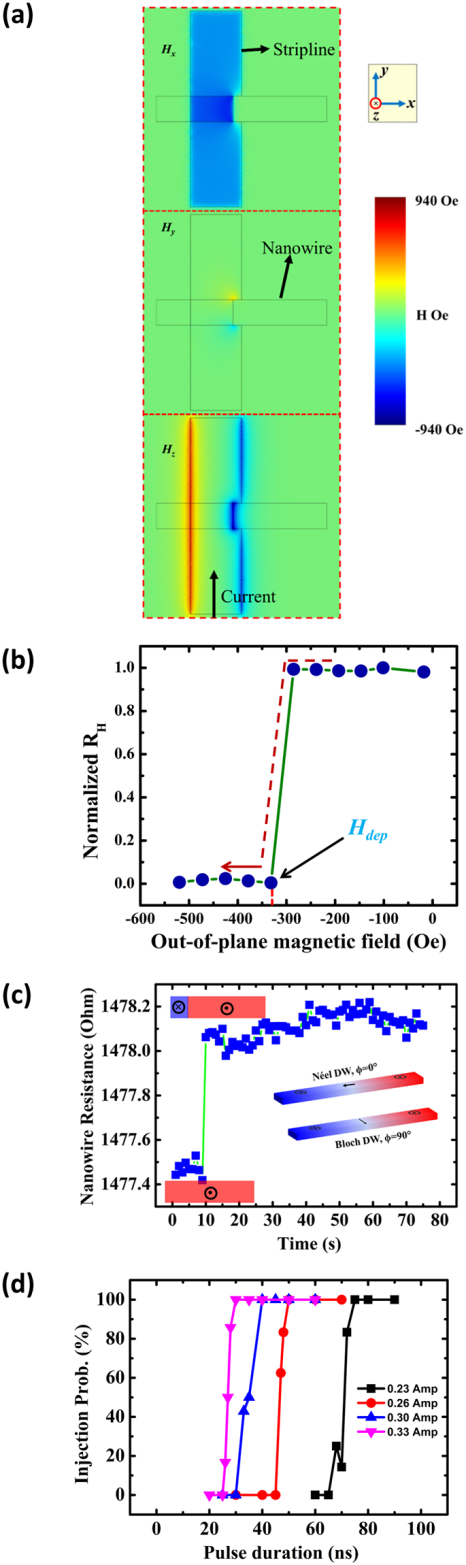
(**a**) COMSOL simulations of the Oersted magnetic field strength in *x, y* and *z* directions for a current density of 6 × 10^11^ A/m^2^. **(b)** Normalized Hall resistance of the nanowire with the negative perpendicular magnetic field sweep, measured after a DW has been injected into a nanowire, saturated along positive *z*-direction. The DW is shown to be depinned from its original position at ~−310 Oe **(c**) Anisotropic magneto-resistance measurements of the nanowire. The change in the resistance concurs the DW injection. Néel and Block walls magnetization profiles are shown in the inset. (**d**) The probability of the DW injection as a function of current pulse duration for various current amplitudes.

First, we examine the relation between the amplitude of the applied current pulse to the duration on the DW injection process. Initially, the nanowire was saturated along the *z*-axis by applying a large global magnetic field > 3 kOe. To inject the DW, current pulses of several amplitudes and duration were applied to the strip line having a width of 2.5 μm and a thickness of 150 nm. The applied current then generates a local Oersted field which nucleates a domain of reversed magnetization under the strip line. Figure 2(a) shows the Oersted field components in *x*, *y* and *z*-direction at the π-shaped strip line and the nanowire interface, as calculated by COMSOL simulations for a current density of 6 × 10^11^ A/m^2^. As shown in Fig. 2(a), the *H*_*x*_ and *H*_*z*_ components of the Oersted field are more confined inside the π–shaped strip line which help to nucleate the DW at that location^14^. After the DW has been injected, an out-of-plane magnetic field in the opposite direction, *i.e*. *–z* direction, was applied and the anomalous Hall effect (AHE) signal was detected at Hall bar-1, simultaneously. The drop in the *R*_*H*_ from 1 to 0 at a magnetic field strength of –310 Oe indicates the successful injection of the DW and termed as the DW depinning field (*H*_*dep*_), as shown in Fig. 2(b). Magneto-resistance measurements were performed in four probe geometry to calculate the DW resistance. As shown in Fig. 2(c), the magneto-resistance of the nanowire was found to be 1477.47 Ω. After the DW has been injected into the nanowire, the resistance was increased to 1478.05 Ω. In the SAF structures, the bottom FM layer is grown on the Pt underlayer that stabilizes Néel DWs due to Dzyaloshinskii-Moriya interaction (DMI)^3,8^. A DW of similar chirality to that of bottom FM layer is stabilized into the upper FM layer due to the antiferromagnetic coupling between the two FM layers. The contribution into magnetoresistance from the DW intrinsic resistance (${R}_{DW\_\mathrm{int}}\propto 1/{{\rm{\Delta }}}^{2}$) is reported negligible (~1 mΩ)^15^ compare to the Néel DWs magnetoresistance (~0.4 Ω)^16^. When current is applied to the nanowire, the dominating contribution into the magneto-resistance arises due to the orientation of Néel DW magnetization parallel to the current flow direction. The contribution in the magneto-resistance from the Néel DWs is proportional to ${\rm{\Delta }}{\cos }^{2}{\rm{\Phi }}$ ^17^. Here *Δ* is the DW width and *Φ* is the in-plane angle between Bloch (Φ = 90°) and Néel (Φ = 0°) DWs state as shown by a schematic in the Fig. 2(c). Therefore, the change in SAF nanowire magneto-resistance ∆R = 0.58 Ω can be attributed to the existence of two antiferromagnetically coupled Néel DWs of width ~25 nm^16,17^. The DW annihilation process is presented in Supp. S2. The probability of DW injection at different current densities with different pulse duration is shown in Fig. 2(d). The result indicates that the probability of DW injection shifts towards lower pulse duration with increase in the current.

**Figure 3:**
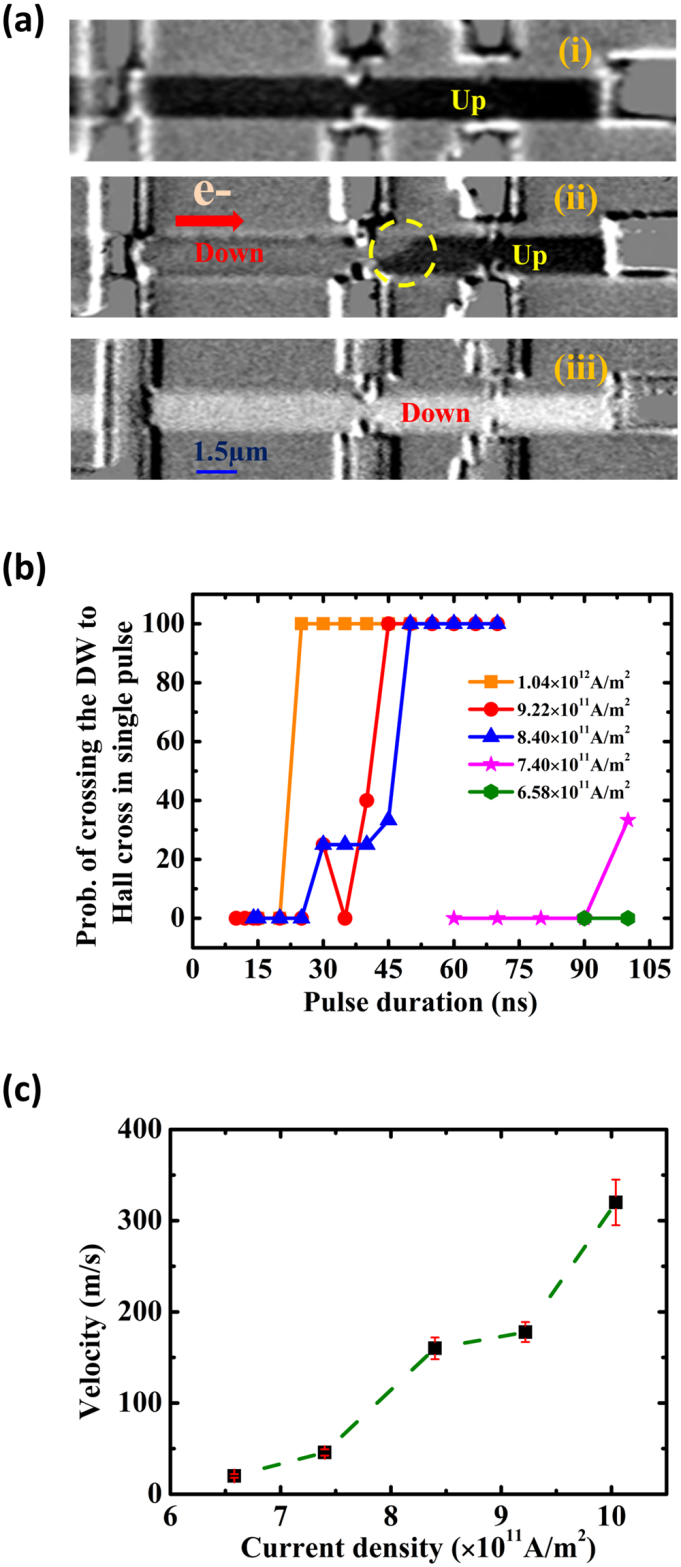
(**a**) Kerr microscopy imaging of DW driving in the SAF nanowire. (i) The nanowire was saturated along the +*z* direction, (ii) a current-driven DW after a successful injection, and (iii) The nanowire was saturated along the −*z* direction. The circled area shows the tilted DW. The directions of the magnetization correspond to bright (Down) and dark (Up) contrast are also labeled. **(b)** Probability of crossing the DW to Hall bar in single pulse at different pulse duration. **(c)** The measured DW velocity at various current densities.

Prior to the DW driving measurements, the DWs were injected using current pulse of 0.3 A and 60 ns. Figure 3(a) shows the Kerr microscopy images of the device. Figure 3(a–i) depicts the magnetic contrast of the nanowire when it was saturated along the + *z-*direction (Up). A DW was then injected and driven by a negative current pulse to Hall bar-1. Two clear domains (Down-Up) can be seen in the Kerr microscopy image as shown in Fig. 3(a–ii). Figure 3(a–iii) shows the Kerr image of the nanowire after it was saturated along the *–z* direction (Down). Two phenomena were observed in Fig. 3(a–ii): first, the motion of DW was along the electron flow direction, *i.e*. against the current flow direction; second, a large tilting of the DW at around ~40° was observed. Boulle’s *et al*.^18^ model suggests that the magnetization tilting of the DW is due to the presence of DMI. The DMI in our Co/Ni SAF stack is attributed by the broken inversion symmetry due to the Pt underlayer and Ta capping layer. The presence of significant DMI in the nanowires forces the DW to be stabilized into Néel configuration^19–22^. Observation of high DW resistance in the SAF nanowires also validates that the DW magnetization was locked into the Néel configuration^16,17^. Both the observation of DW tilting in the Kerr microscopy and the large DW resistance revealed the stabilization of Néel DWs over Bloch DWs in our SAF stack.

On the other hand, the direction of DW motion depends on the chirality of the stabilized Néel DW^5^. It is clear from our Kerr microscopy imaging as discussed in Fig. 3(a) that the DW motion in our SAF stack was along the electron flow direction. When current was passed through the nanowire, spin currents generated by the SHE at the top and bottom interfaces exerted a torque on the Néel DWs. The SHE torque is Slonczewski-like in nature and given by:

1 $${\overrightarrow{\tau }}_{SHE}=-{\gamma }_{0}\overrightarrow{m}\times (-\frac{\hslash {\theta }_{SH}{j}_{a}}{2{\mu }_{0}|{e}^{-}|{M}_{s}t}\overrightarrow{m}\times {\overrightarrow{u}}_{y})=-{\gamma }_{0}\overrightarrow{m}\times {\overrightarrow{H}}_{SHE},$$

where *J*_*a*_ is the applied current density in *−x* direction, *M*_*s*_ is the saturation magnetization, *t* is the thickness of magnetic layer, $\overrightarrow{m}$ is the magnetization vector of the DW, and ${\overrightarrow{u}}_{y}$ is a unit vector in *y-*direction, *θ*_*SH*_ is the effective spin Hall angle, |*e* ^*−*^| is the absolute value of the electron charge. Although Ta and Pt have opposite signs of *θ*_*SH*_^2,5^, but an enhanced SHE is obtained due to their position at opposite interfaces^23,24^. Thus, the sign of the net *θ*_*SH*_ of our SAF is similar to that of Pt, *i.e. θ*_*SH*_ > 0. From eq. 1, the direction of the SHE torque is directed along the *y-*direction which results in the spin rotation towards the nanowire transverse axis. The term in the bracket is the spin Hall effective magnetic field (*H*_*SHE*_), whereby:

2 $${\overrightarrow{H}}_{SHE}=-\frac{\hslash {\theta }_{SH}{j}_{a}}{2{\mu }_{0}|{e}^{-}|{M}_{s}t}\overrightarrow{m}\times {\overrightarrow{u}}_{y},$$

From equation 2, it can be concluded that *H*_*SHE*_ depends on the magnetization of the DW and determines the direction of the DW motion. The directions of *H*_*SHE*_ with respect to the magnetic domains and the DWs chirality are shown in a schematic in Supp. S3.

For the bottom FM layer, the SHE mainly comes from the Pt/FM interface, and thus the effective field *(H*_*SHE*_)_*b*_ that acts on the bottom DW can be written as:

3 $${({\overrightarrow{H}}_{SHE})}_{b}=-\frac{\hslash {\theta }_{SH}{j}_{a}}{2{\mu }_{0}|{e}^{-}|{M}_{s}t}\overrightarrow{m}\times {\overrightarrow{u}}_{y}=\frac{\hslash {\theta }_{SH}{j}_{a}}{2{\mu }_{0}|{e}^{-}|{M}_{s}t}({\overrightarrow{m}}_{x})\times {\overrightarrow{u}}_{y}=\frac{\hslash {\theta }_{SH}{j}_{a}}{2{\mu }_{0}|{e}^{-}|{M}_{s}t}(\hat{z}).$$

Here, we assume that magnetization of the Néel DW in the bottom FM layer points into the + *x* direction due to the DMI. The effective field generated by the SHE is in the positive out-of-plane direction (+z), which helps to grow the “Up” domain and results in the DW propagating along the positive *x*-direction. Because of the AFM coupling, magnetization of the DW in the top FM layer is pointed along the –*x* axis. The effective field on the top DW *(H*_*SHE*_)_*t*_ is given by:

4 $${({\overrightarrow{H}}_{SHE})}_{t}=-\frac{\hslash {\theta }_{SH}{j}_{a}}{2{\mu }_{0}|{e}^{-}|{M}_{s}t}\overrightarrow{m}\times {\overrightarrow{u}}_{y}=\frac{\hslash {\theta }_{SH}{j}_{a}}{2{\mu }_{0}|{e}^{-}|{M}_{s}t}(-{\overrightarrow{m}}_{x})\times {\overrightarrow{u}}_{y}=\frac{\hslash {\theta }_{SH}{j}_{a}}{2{\mu }_{0}|{e}^{-}|{M}_{s}t}(-\hat{z}).$$

The effective field generated by the SHE is in the negative out-of-plane direction, which favours the growth of “Down” domain and results in the DW propagating along the positive *x*-direction. To illustrate the direction of the SHE fields in both layers, a schematic figure is included in Supp. S3.

From equations 2–4, the effective DMI from the top and bottom heavy metal layers can be inferred to lock the DW internal magnetization into the Néel DW with right-handed chirality^5^, and the DW move into the electron flow direction as observed in our experiments. To study the current-induced DW dynamics, current pulses of different amplitude and pulse duration were applied to the nanowire. The DW motions were detected at Hall bar-1 by using the AHE measurement. Each measurement was repeated 10 times at zero magnetic field. The probability of driving the DW beyond the Hall bar-1 in single pulse is plotted in Fig. 3(b). As we increase the current density, the probability of driving the DW beyond the Hall bar-1 in single pulse increases. As expected, less time is required for the DW to cross the Hall bar for higher current densities. Here, as noted from Equation 2, *H*_*SHE*_ increases with higher current density, which results in higher DW velocity. Also, the conventional spin transfer torque (STT) acts in the same direction and assists the DW motion in the direction of electron flow. The DW velocity for different current densities is then calculated using the pulse duration at which the probability of reaching the DW at Hall bar is at 100%, as shown in Fig. 3(c). The threshold current density for our stack was found to be 6.58 × 10^11^ A/m^2^. The DW velocity at current density of 1.04 × 10^12^ A/m^2^ was found to be ~320 m/sec. The higher DW speed in the SAF nanowires is attributed to the presence of the enhanced RKKY interlayer exchange torque due to the SHE induced perturbation in DWs antiferromagnetic coupling.

### Micromagnetic Simulations

**Figure 4:**
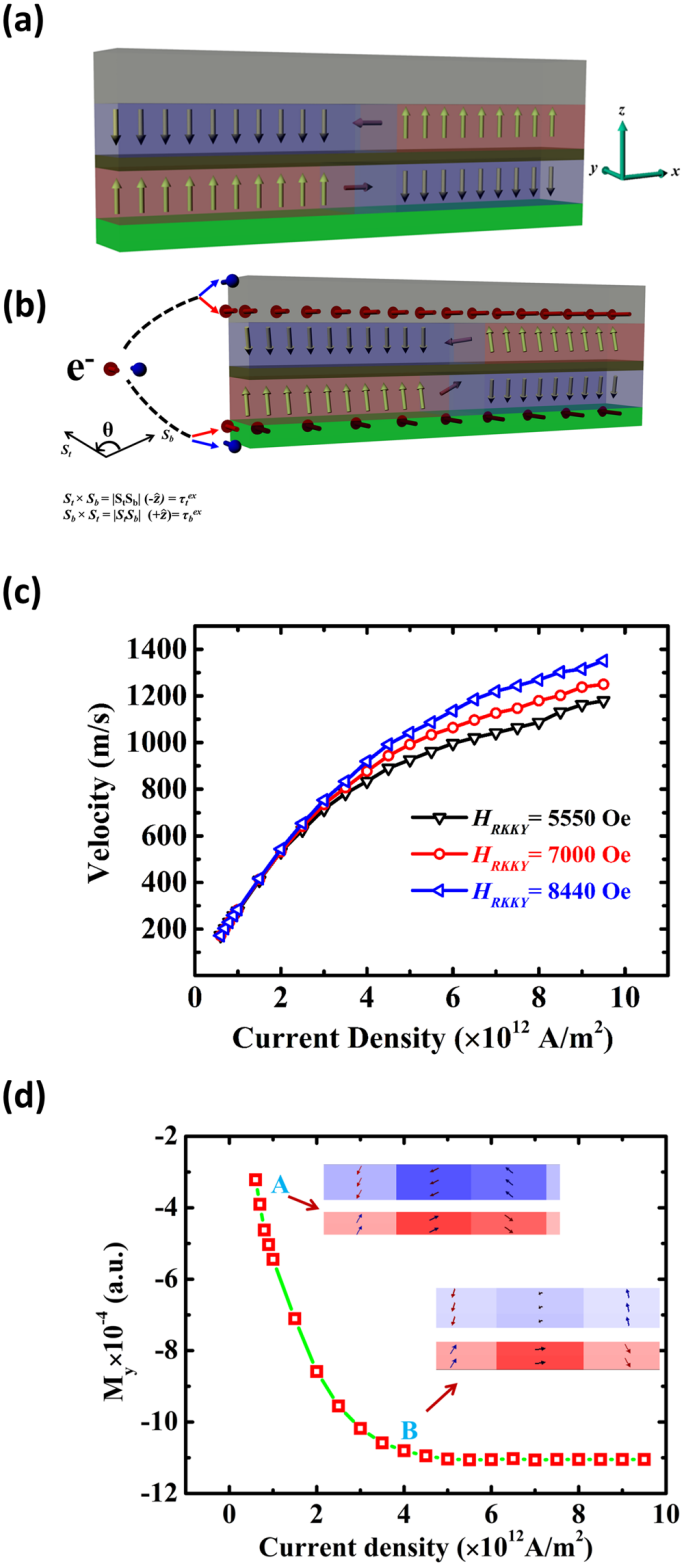
(**a**) Schematic diagram of the SAF nanowire at t = 0 sec and *J*_*a*_ = 0, employed in our micromagnetic model. Both the layers are coupled in antiferromagnetic manner. A DW is nucleated at the middle of the nanowire. **(b)** The schematic diagram of the spin configurations in SAF nanowire at t = *x* sec and *J*_*a*_≠0. Scattering of spin currents (red and blue arrows) in top Ta and bottom Pt layers. **(c)** Plot of the DW velocities as a function of current density for various RKKY exchange coupling strengths. **(d)** The *y*-component of the DW magnetization as a function of current densities. The side-view simulation snapshots at low current density (point ‘A’) and high current density (point ‘B’) are also shown.

To understand the effect of RKKY interlayer exchange coupling on the DW dynamics, the interlayer RKKY exchange torque (*τ*_*RKKY*_) and SHE torque terms (*τ*_*SHE*_) were added into the Landau-Lifshitz-Gilbert (LLG) equation. Mumax micromagnetic simulations were performed^25^ and the details of simulation methods are presented in methods section. Figure 4(a) shows a schematic diagram of the SAF nanowire, in which an antiferromagnetically coupled Néel DW was nucleated at the center of the nanowire. Spin polarized currents of various amplitudes were then applied to the nanowire along the *x*-axis direction. The scattered spin currents from the bottom Pt (*θ*_*SH*_ > 0) and the top Ta (*θ*_*SH*_ < 0) layers are shown by the red arrows at the Pt/FM and FM/Ta interfaces in Fig. 4(b). The accumulated spin currents induce a torque on the FM layers and the DWs towards the nanowire transverse direction, *i.e*. + *y* direction. The simulated DW velocity for different RKKY interlayer AFM couplings is shown in Fig. 4(c). The contribution of the RKKY exchange torque can then be seen from the increases of the DW velocity with larger RKKY antiferromagnetic exchange strength. As shown in Fig. 4(c), for a fixed current density ~8 × 10^12^ A/m^2^, the DW velocity is increased by ~190 m/s when *H*_*RKKY*_ was increased from 5550 Oe to 8440 Oe. Our calculations show that the magnitude of *τ*_*RKKY*_ depends on the RKKY coupling strength as well as the cross product of the DWs spins in the top and bottom layer. The RKKY torque on a DW in bottom (top) FM layer due to another DW in top (bottom) FM layer can be given by-

5 $${\tau }_{b(t)}^{RKKY}=-{\overrightarrow{m}}_{b(t)}\times {\overrightarrow{B}}_{b(t)}^{RKKY}=-2J({\overrightarrow{S}}_{b(t)}\times {\overrightarrow{S}}_{t(b)}).$$

Here, *S*_*b*_ and *S*_*t*_ are the spins into bottom and top FM layers, respectively. The detailed analysis of *τ*_*RKKY*_ and its direction are added into Supp. S4. The *τ*_*RKKY*_ is zero in the absence of current as both the DWs were perfectly antiparallel to each other. When current is applied, both the DWs are rotated in the same direction (+*y* direction) and are perturbed from their antiparallel coupling due to the torque from the SHE according to Equation 1. Our simulation results showed that the perturbation increases with higher current densities due to the larger SHE, and both the DWs were perpendicular to each other at higher current densities. The magnitude of *τ*_*RKKY*_ was maximum when both the DWs were perpendicular to each other. The *τ*_*RKKY*_ functioned in such a way that both the DWs were driven in electron flow direction. The DW velocity saturates at higher current densities for all RKKY interlayer exchange strengths, as shown in Fig. 4(c). Figure 4(d) shows the average *M*_*y*_-component of both the DWs for different current densities. The plot shows that the angle between the two DWs is fixed at higher current density, which results in the saturation of the exchange torque and the DW velocity.

**Figure 5:**
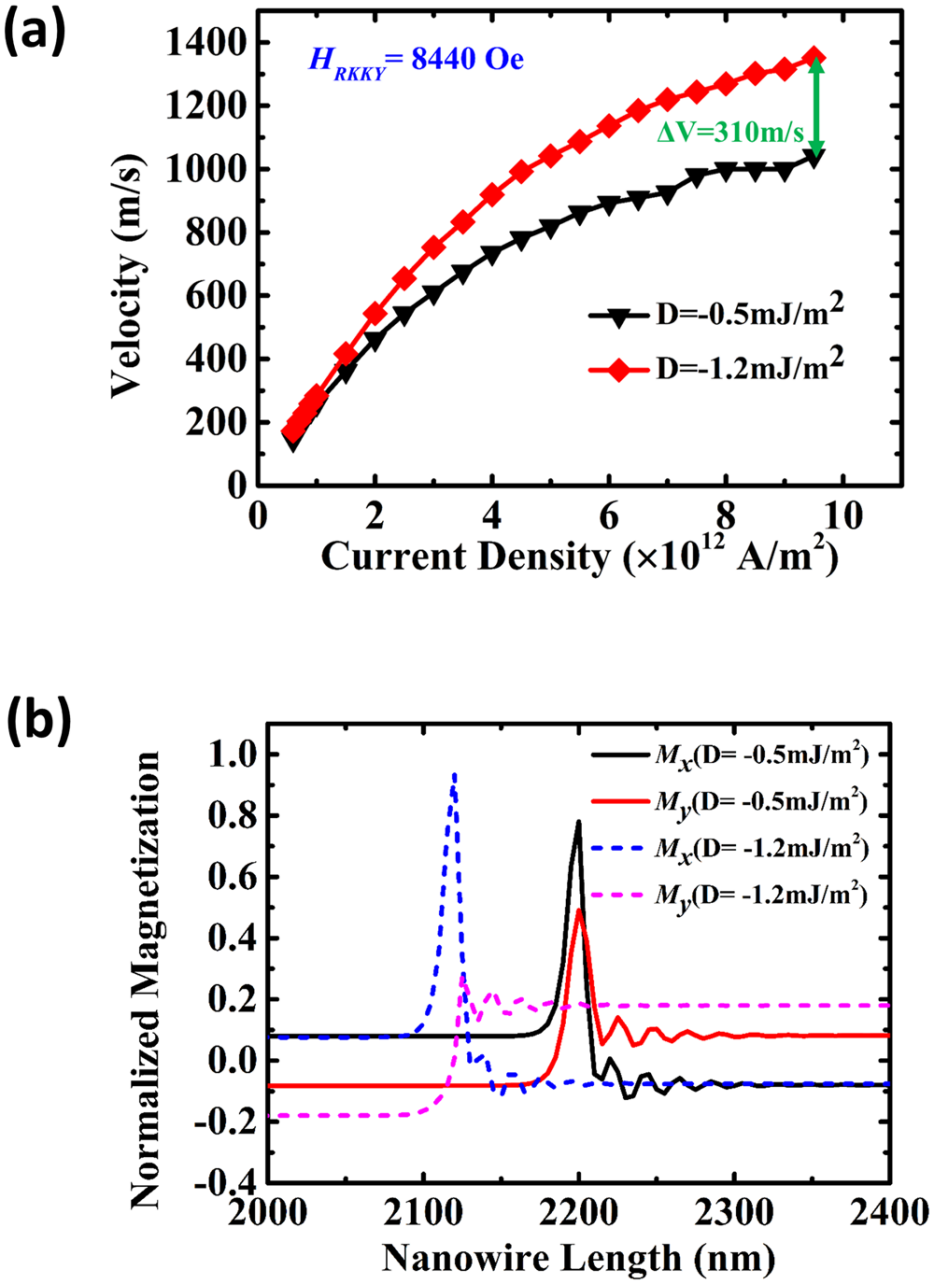
(**a**) Domain wall velocity as a function of current density for two different DMI values −0.5 mJ/m^2^ (black) and −1.2 mJ/m^2^(red). **(b)** Normalized *x* and *y* components of the DW magnetization at 0.3 ns for DMI (D) = −0.5 mJ/m^2^ (solid lines) and −1.2 mJ/m^2^ (dash lines).

Figure 5(a) shows the DW velocity as a function of current density for two different DMI values −0.5 mJ/m^2^ (black) and −1.2 mJ/m^2^ (red). A significant increment in the DW velocity ~310 m/s is observed at a current density of 9 × 10^11^ A/m^2^, when the DMI is increased from −0.5 mJ/m^2^ to −1.2 mJ/m^2^. Higher values of the DMI is shown to increase the *M*_*x*_ components of the DW which gives higher DW speeds due to the spin Hall effect. Figure 5(b) shows the normalized *x* and *y* components of the DW magnetization for two DMI values: −0.5 mJ/m^2^ (solid lines) and −1.2 mJ/m^2^ (dash lines). The increase in *x*-component with the higher DMI value indicates the stabilization of the Néel DW. The results show that nanowires with high DMI values will be helpful for the realization of high speed magnetic memory devices.

## Conclusion

In conclusion, we have investigated the DW injection, the current-induced driving, and the electrical detection in perpendicularly magnetized SAF nanowires. Observation of DW tilting in addition to high DW resistance is attributed to the presence of DMI from the Pt underlayer and the Ta capping layers. The DMI from the heavy-metal/FM interfaces locks the DW spins into Néel configuration. The SHE-induced torque, generated by placing heavy-metals of opposite signs at the bottom and the top FM interfaces, allows for efficient DW motion along the electron flow direction. Furthermore, the SHE-induced perturbation in the antiferromagnetic coupling of the DWs stimulates an enhanced interlayer exchange torque at low current densities, and the current-induced DW motion was observed with velocity larger than 300 m/s at 1.04 × 10^12^ A/m^2^. Micromagnetic simulations confirm the experimental results and allow us to explain the interplay between the SHE and the exchange torque on the DW dynamics. The high speed DW motion at relatively low current densities will provide a helpful design for high speed magnetic memory and logic devices^1,3–5,10,26–30^.

## Methods

### Thin Film deposition

Ta(3)/Pt(3)/[Co(0.4)/Ni(0.7)/Co(0.4)]/Ru(0.8)/[Co(0.4)/Ni(0.7)/Co(0.4)]/ Ta(3) thin film stack was deposited on thermally oxidized SiO_2_ substrate using DC magnetron sputtering techniques. Numbers in parenthesis represent the layer thickness in ‘nm’. In the thin film stack, two magnetic trilayer (bottom stack −M_1_ and top stack −M_2_) structures of Co(0.4)/Ni(0.7)/Co(0.4) are coupled through a Ru spacer layer. A vibrating sample magnetometer (VSM) was used to measure hysteresis behavior of the magnetic thin film.

### Device fabrication

First, the thin film stack was coated with negative resist and patterned using electron-beam lithography and Ar ion milling technique. Second, the nanowires were spin coated with positive resist to pattern electrical contacts. Third, the Ta/Cu/Au electrodes were deposited using magnetron sputtering after a reverse sputtering process to ensure good Ohmic contacts. Last, Lift-off of the metallic film was completed in acetone to obtain the final device. The length and width of fabricated nanowires were 30 µm and 1.5 µm, respectively

### Electrical measurements

A picosecond pulse generator (Picosecond 10300B) was used to inject the DWs by applying a current pulse to the π-shaped Ta/Cu/Au strip line (contact A → B) by generating local Oersted field. The injected DWs were then driven by applying electrical pulses (I_STT_) of different amplitudes and duration between contacts A and C of the device. Two Hall bars were also patterned on the nanowire to detect the DWs by using anomalous Hall effect (AHE). A Keithley 2400 DC current source was used to supply a low amplitude current density (I_READ_ = 6 × 10^9^A/m^2^) between contacts ‘A’ and ‘C’ to measure the Hall voltage across the Hall bar-1 and magneto-resistance of the nanowire. A Keithley 2000 voltmeter was also connected between the contacts A and C to measure the magneto-resistance of the nanowire. The spacing between the strip line and the Hall bar-1, which served as the primary DW detector, was kept as 8 µm. All the electrical measurements were performed on a 40-GHz Cascade Microtech probe station.

### Micromagnetic simulations

The general form of the Landau-Lifshitz-Gilbert (LLG) equation with the spin transfer torque (STT) term can be written by

6 $$\frac{\partial \overrightarrow{m}}{\partial t}=-{\gamma }_{0}\overrightarrow{m}\times {\overrightarrow{H}}_{eff}+\alpha \overrightarrow{m}\times \frac{\partial \overrightarrow{m}}{\partial t}+{\overrightarrow{\tau }}_{STT}$$

The first term in the above equation is the precession term. Where *γ*_0_ is the gyromagnetic ratio and *H*_*eff*_ is the effective field. The second term is damping term with Gilbert damping constant ‘α’. The third term is the Zhang-Li spin transfer torque term that includes both adiabatic and non-adiabatic contributions. The adiabatic STT deforms the DW magnetization and only crucial for the initial DW motion. While the non-adiabatic STT acts as a non-uniform magnetic field and controls the DWs terminal velocity. The modified LLG equation^31^ is expressed as:

7 $$\frac{\partial \overrightarrow{m}}{\partial t}=-{\gamma }_{0}\overrightarrow{m}\times {\overrightarrow{H}}_{eff}+\alpha \overrightarrow{m}\times \frac{\partial \overrightarrow{m}}{\partial t}-u\overrightarrow{m}\times \frac{\partial \overrightarrow{m}}{\partial x}\times \overrightarrow{m}+\xi u\overrightarrow{m}\times \frac{\partial \overrightarrow{m}}{\partial x},$$

where, $$u=-\frac{PJ{\mu }_{B}}{e{M}_{s}(1+{\xi }^{2})}$$

In the above equation, *ξ* is the degree of non-adiabaticity, and *P* is the spin-polarization.

The above LLG equation was then modified by adding three additional terms: (1) RKKY interlayer exchange term and (2) SHE induced torque term (3) Dzyaloshinskii-Moriya Interaction term.

### RKKY interlayer exchange term

A modified RKKY exchange field term is included in the effective field (*H*_*eff*_) term. Instead of the usual 6 nearest-neighbor small-angle approximation for exchange interaction^32^, the influence from the next nearest top and bottom magnetic moment was also considered. The modified algorithm allows for the calculation of the exchange coupling between two ferromagnetic materials even when they sandwich a non-magnetic Ruthenium (Ru) spacer. The exchange field that a moment ‘*m*’ experiences due to its neighbor ‘*m*_*i*_’ is given by:

8 $${\mathop{B}\limits^{\rightharpoonup }}_{RKKY}=2\frac{{A}_{ex}}{{M}_{sat}}\sum _{i}{C}_{i}\frac{({\mathop{m}\limits^{\rightharpoonup }}_{i}-\mathop{m}\limits^{\rightharpoonup })}{{{{\rm{\Delta }}}_{i}}^{2}},$$

where *A*_*ex*_ is the exchange stiffness, *M*_*sat*_ is the saturation magnetization, Δ_i_ is the separation distance between the two moments, and *C*_*i*_ is an arbitrary scaling factor which determines the strength of the RKKY interaction and is equals to 1 for nearest neighbors^25^.

### Spin Hall effect torque term

Due to the presence of heavy metals such as Ta and Pt, an in-plane current produces two forms of torque on the magnetization; the spin transfer torque that is modeled as a Zhang-Li spin torque *τ*_*STT*_ according to equation 6 and a spin Hall torque *τ*_*SL*_ that is modelled by a Slonczewski spin-transfer torque. The LLG equation is thus modified to consider Slonczewski spin-transfer torque (τ_SL_):

9 $$\frac{\partial \overrightarrow{m}}{\partial t}={\overrightarrow{\tau }}_{LL}+{\overrightarrow{\tau }}_{STT}+{\overrightarrow{\tau }}_{SL},$$

Here

10 $${\overrightarrow{\tau }}_{SL}=-{\gamma }_{0}\overrightarrow{m}\times (-\frac{\hslash {\theta }_{SH}{j}_{a}}{2{\mu }_{0}|{e}^{-}|{M}_{s}t}\overrightarrow{m}\times {\overrightarrow{u}}_{y}),$$

Where *θ*_*SH*_ is the spin Hall angle, *j*_*a*_ is the applied current density, *t* is the thickness of the magnetic volume considered and *u*_*y*_ is an in-plane unit vector that points in the *y*-direction.

### Dzyaloshinskii-Moriya Interaction term

The DMI exchange field term is included in the effective field (*H*_*eff*_) term of the LLG equation. The DMI exchange energy (*E*_*ij*_) between two spins ‘*S*_*i*_’ and ‘*S*_*j*_’ can be given by

11 $${E}_{ij}={\overrightarrow{D}}_{ij}\bullet ({\overrightarrow{S}}_{i}\times {\overrightarrow{S}}_{j}).$$

Here, *D*_*ij*_ is the DM interaction vector and its direction depends on the studied system. For the FM thin films that are grown on heavy metals of high spin-orbit coupling, the DMI constant is given by

12 $${\overrightarrow{D}}_{ij}=d\,{\overrightarrow{u}}_{ij}\times \hat{z},$$

where, *u*_*ij*_ is the unit distance vector between the two spins *S*_*i*_ and *S*_*j*_, and $\hat{z}$ is the unit vector perpendicular to the thin film plane from heavy metal to FM thin film. Even though, the DMI is interfacial phenomenon, a uniform value of DMI can be considered along the SAF thin film thickness (t) and the DMI energy can be written as^33,34^:

13 $${E}_{DM}=t\iint D[({m}_{x}\frac{\partial {m}_{z}}{\partial x}-{m}_{z}\frac{\partial {m}_{x}}{\partial x})+({m}_{y}\frac{\partial {m}_{z}}{\partial y}-{m}_{z}\frac{\partial {m}_{y}}{\partial y})]{d}^{2}\overrightarrow{r}$$

Here, *D* is the continuous DMI constant across the thin film thickness. The SAF structures are formed of a bottom FM layer that is coupled with an upper FM layer through RKKY exchange coupling. Since the bottom FM layer is grown on the Pt, the DWs in the bottom FM layer experience an additional local field, the DMI field ${\overrightarrow{H}}_{DM}=-\frac{\partial {E}_{DM}}{\partial \overrightarrow{M}}$. The direction of the DMI field is collinear to the *u*_*ij*_ that is along the *x*-direction. The DMI field stabilize the DWs into Néel configuration with a preferred chirality. The contribution in the magnetization dynamics from the *H*_*DM*_ has been included into the effective field terms (H_eff_) of the LLG equation. In the micromagnetic simulations, the DMI fields were considered similar for both the layers and the DWs velocities were calculated for two DMI values (D) = −0.5 mJ/m^2^ and −0.5 mJ/m^2^.

The chosen material parameters were initially set to: saturation magnetization (*M*_*s*_) = 6 × 10^5^ A/m, exchange stiffness constant (*A*_*ex*_) = 1.3 × 10^−11^ J/m, damping constant (α) = 0.1, non-adiabaticity of STT (ξ) = 0.35^7^, spin Hall angle (*θ*_*SH*_) = 0.2 and DMI (D) = −1.2 × 10^−3^ J/m^2^ & D = −0.5 × 10^−3^ J/m^2^ ^33^. A mesh size of 5 × 5 × 0.8 nm^3^ was used throughout this work. The thicknesses of the bottom and the top ferromagnetic layers were fixed at 1.6 nm and 2.4 nm, respectively. Both the FM layers were coupled via an antiferromagnetic coupling through a 0.8 nm thick Ru spacer layer. The antiferromagnetic coupling strength was varied correspond to three different exchange fields (*H*_*RKKY*_): 8440 Oe, 7000 Oe and 5550 Oe.

## Electronic supplementary material

Supplementary information
