# Supplementary material for: Role of RKKY torque on domain wall motion in synthetic antiferromagnetic nanowires with opposite spin Hall angles
Source: Sci Rep. 2017 Sep 15;7:11715. doi: 10.1038/s41598-017-11733-9 (PMC5601456; doi:10.1038/s41598-017-11733-9)
Supplement: Supplementary file 1 — Supplementary information [file 41598_2017_11733_MOESM1_ESM.pdf]

Supplementary information

Role of RKKY torque on domain wall motion in synthetic antiferromagnetic nanowires with opposite spin Hall angles

S. Krishnia[1], P. Sethi[1], W. L. Gan[1], F. N. Kholid[1], I. Purnama[1], M. Ramu[1], T. S. Herng[2], J. Ding[2] and W. S. Lew[1]*

[1]*School of Physical and Mathematical Sciences, Nanyang Technological University, 21 Nanyang Link, Singapore 637371*
[2]*Department of Materials Science and Engineering, National University of Singapore, 9 Engineering Drive 1, Singapore 117576*

Supplementary 1 – Growth of synthetic antiferromagnetic (SAF) stack with perpendicular magnetic anisotropy (PMA).

Fig. S1(a) and S1(b) show the Magneto Optic Kerr Microscopy (MOKE) hysteresis loop of Ta(3)/Pt(3)/Co(0.4)/Ni(0.7)/Co(0.4)/Ta(3) and Ta(3)/Pt(3)/[Co(0.4)/Ni(0.7)]$_2$/Co(0.4)/Ta(3) that were grown by DC magnetron sputtering with coercivity of ~55 Oe and ~110 Oe, respectively. A Ru spacer layer was inserted between the two Co/Ni/Co trilayer structures to introduce the RKKY antiferromagnetic coupling to obtain a synthetic antiferromagnetic structure. VSM measurements were carried out to measure the easy and the hard axis anisotropy of the SAF, and the results are shown in Fig. S1(c). The black and red hysteresis correspond to in-plane and out-of-plane magnetic field sweeps, respectively. The easy axis anisotropy was found to 380 Oe while the hard axis anisotropy field was found to be ~5.5kOe.

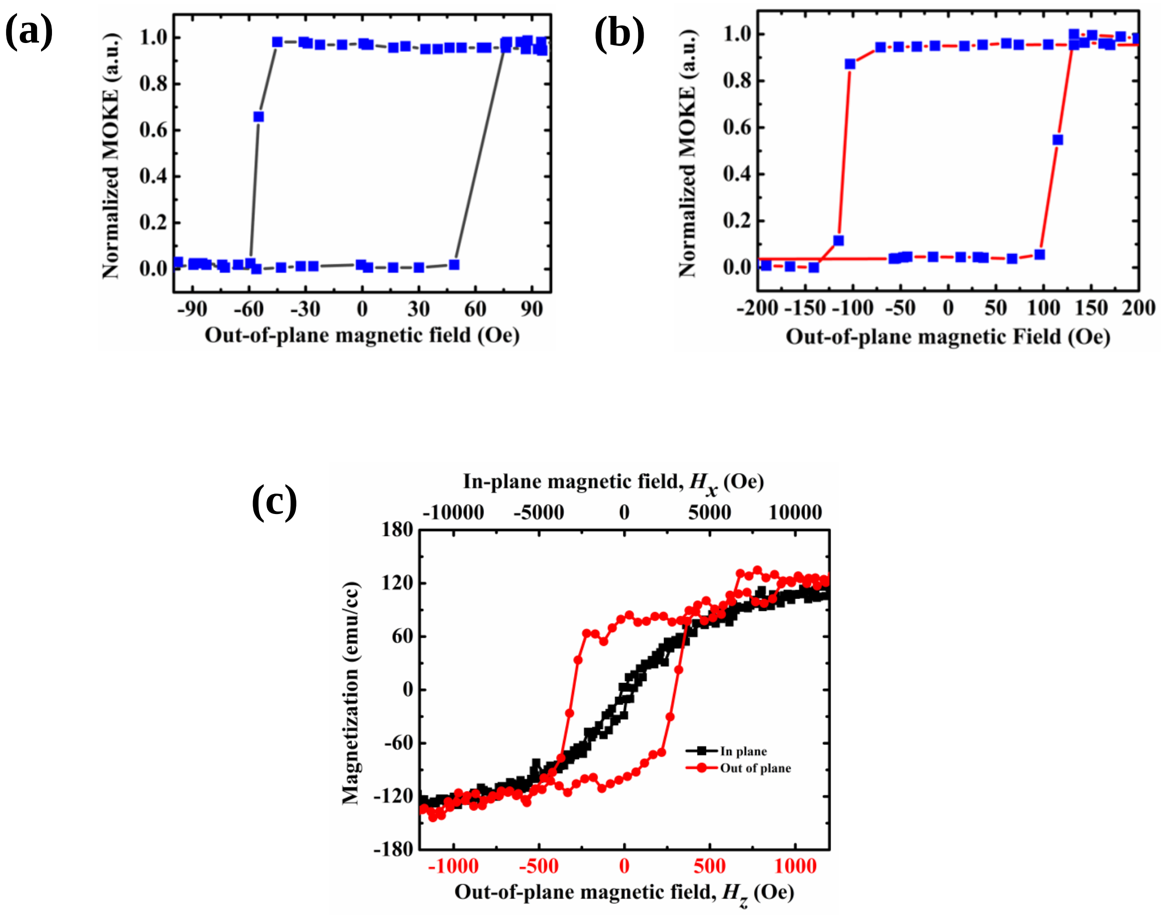

Fig. S1 **(a)** MOKE Hysteresis of Ta(3)/Pt(3)/Co(0.4)/Ni(0.7)/Co(0.4)/Ta(3) and **(b)** Ta(3)/Pt(3)/[Co(0.4)/Ni(0.7)]$_2$/Co(0.4)/Ta(3) thin film stacks **(c)** In-plane (black) and out-of-plane (red) hysteresis loops of Ta(3)/Pt(3)/[Co(0.4)/Ni(0.7)/Co(0.4)]/Ru(0.8)/[Co(0.4)/Ni(0.7)/Co(0.4)]/Ta(3) SAF stack measured from VSM.

Supplementary 2 – Injection and annihilation of the DWs and its motion around the Hall cross.

Fig. S2(a) shows the anisotropic magneto-resistance (AMR) measurements of the SAF nanowire. The change in the AMR (ΔR) represents the DW resistance (R_{DW}). The AMR regains its primary value when the DW is annihilated by applying opposite current pulse to the injection line. Fig. S2(b) shows the back-and-forth current-induced DW motion around the Hall bar. After

the DW was injected, it was driven by a negative current pulse to Hall bar-1 and a change in Hall signal from ‘1’ to ‘0’ was detected. The DW was then driven back with a positive current pulse and a rise from ‘0’ to ‘1’ in the Hall was detected. The switching between ‘0’ and ‘1’ in the Hall resistance with opposite current pulses reveals that the DW can be driven in both directions by changing the current polarity.

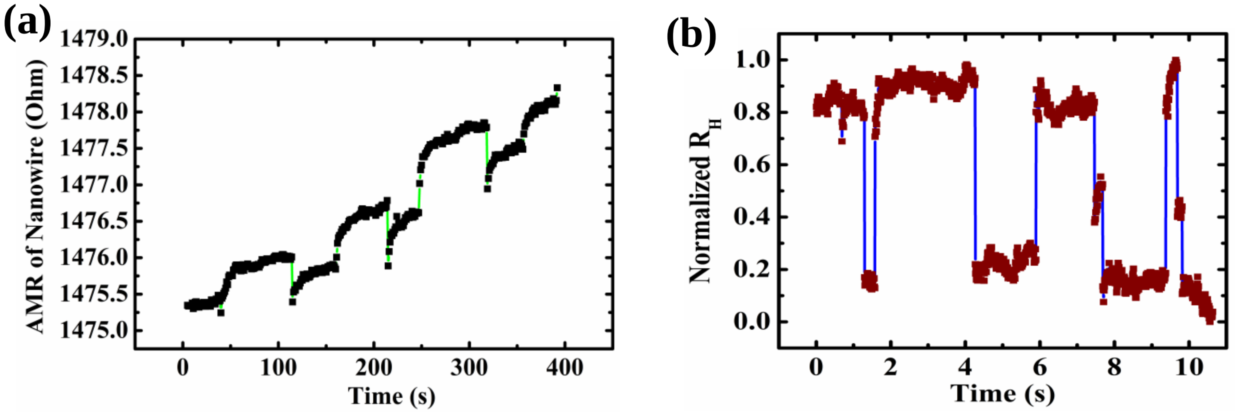

Fig. S2 **(a)** Injection and annihilation of the DW in SAF nanowire. The jump in the anisotropic magneto-resistance (AMR) corresponds to the injection of the DW. The nanowire’s AMR regains its primary value after annihilating the DW by applying opposite current pulse to the injection line. **(b)** Detection of the back-and-forth motion of the DW by anomalous Hall Effect.

Supplementary 3 –Direction of Spin Hall Effect (SHE) effective field.

We assume that ‘Down-Left-Up’ Néel DW was stabilized in the top layer. The DW is called ‘Down-Left-Up’ when the magnetization of DW is pointed along the $-x$ direction as shown in the schematic of Fig S3. Because of the antiferromagnetic coupling, ‘Up-Right-Down’ Néel DW was stabilized in the bottom layer. The DW is called ‘Up-Right-Down’ when the magnetization of DW is pointed along the $+x$ direction. When the spin polarized currents were applied to the nanowire, effective magnetic fields were generated in both the nanowires due to the SHE; this results in the ‘Down-Left-Up’ and the ‘Up-Right-Down’ DWs to move in the

electron flow direction. The magnetic domains and DWs in the nanowire and the direction of effective SHE field on the DWs are shown by a schematic in Fig. S3.

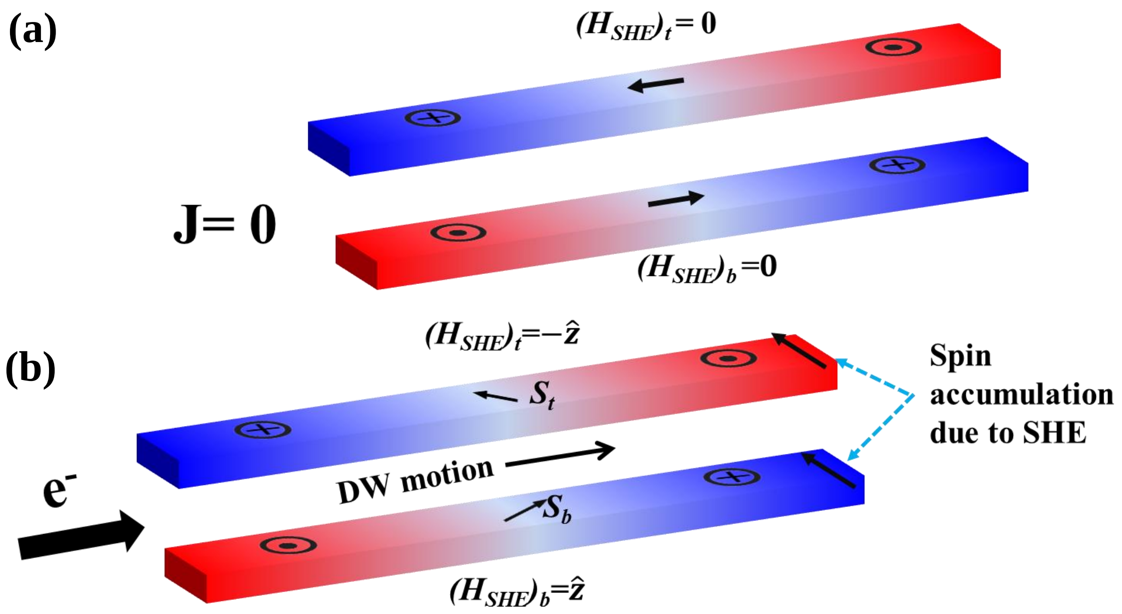

Fig. S3 Schematics of the SAF nanowire; showing the magnetic domains and DWs magnetization directions in the **(a)** absence and **(b)** presence of the current. The direction of SHE induced magnetic fields are also shown.

Supplementary 4 –Direction of RKKY exchange torques

Fig. S4 shows the schematic diagrams of the DW configurations in the absence (a) and presence (b) of the current. Our nanowires were composed of two ferromagnetic (FM) layers which were coupled antiferromagnetically. In top FM layer a 'Down-Up' Néel DW was nucleated while in bottom FM layer the 'Up-Down'. In the absence of current density, Néel type DWs were stable because of the high DMI. During the application of current, the two DWs are both rotated into +*y* direction which makes them to be non-collinear, which further stimulates the RKKY exchange torque, as shown in the schematic of Fig. S4(b).

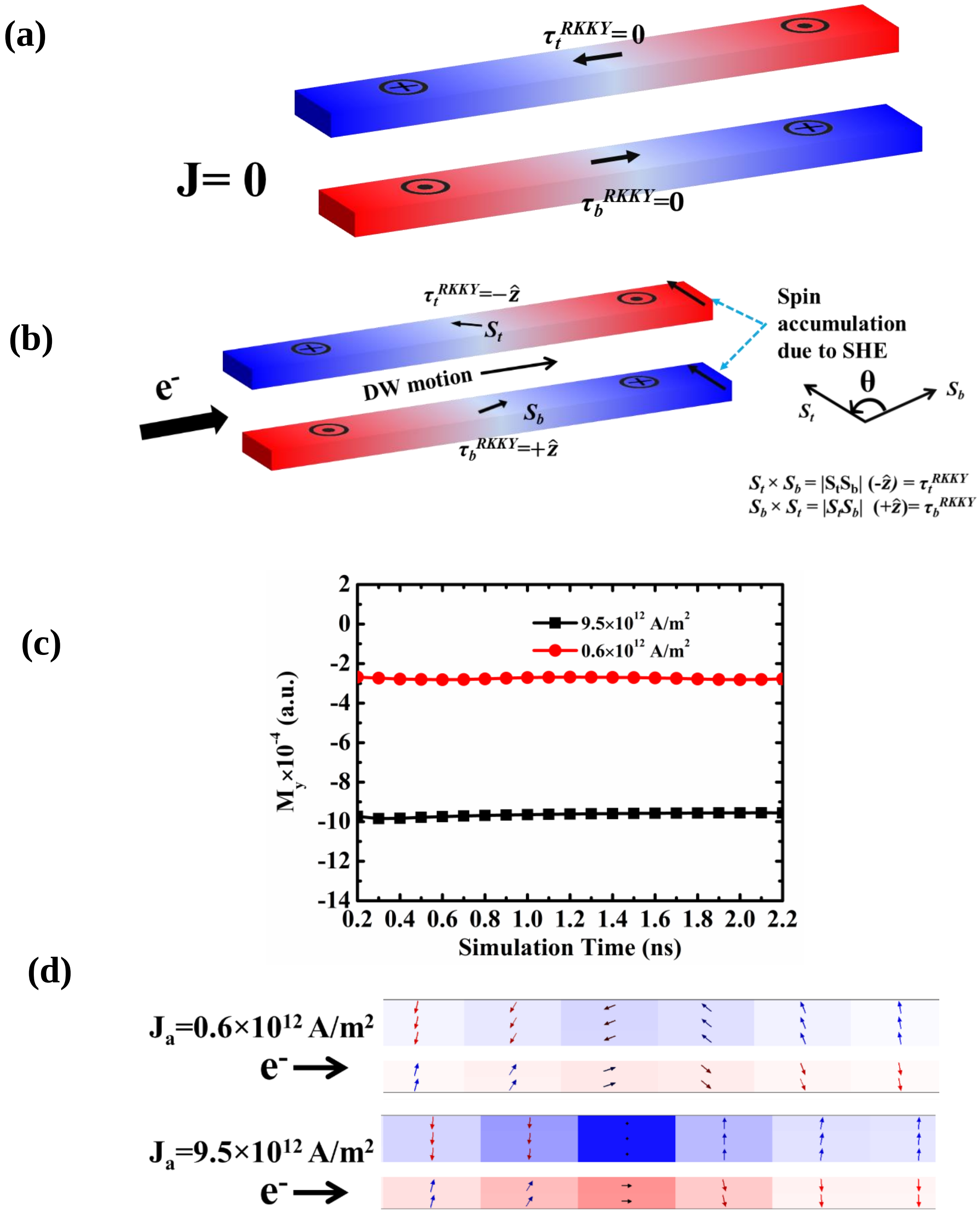

Fig. S4 Schematics showing the RKKY exchange torque directions on the DWs in the **(a)** absence and **(b)** presence of current. **(c)** Comparison of 'M_y' components of DWs at high and low current densities. **(d i-ii)** Simulation snapshots at respective current densities.

The exchange torques on the DWs can be determined by calculating the exchange energy (E_{ex}) between spins of the top DW (S_t) and the bottom DW (S_b):

$$E_{ex} = -2J\vec{S}_t \cdot \vec{S}_b = -2JS_tS_b\cos\theta \qquad \begin{cases} J < 0; \text{for antiferromagnetic coupling} \\ J > 0; \text{for ferromagnetic coupling} \end{cases} \tag{1}$$

Where 'θ' is angle between the spins of two DWs and J is exchange coupling constant. From the exchange theory of two spins, the magnetic moment of top DW spin can be written as $\vec{m}_t = -g\mu_t\vec{S}_t$. The magnetic field experienced (B_t^{RKKY}) by the top DW can then be expressed as [R1]:

$$\vec{B}_t^{RKKY} = -\frac{\partial E^{ex}}{\partial m_t} = -\frac{2J}{g\mu_t}\vec{S}_b, \tag{2}$$

Hence, the torque on the top DW due to the exchange field is given by:

$$\tau_t^{RKKY} = -\vec{m}_t \times \vec{B}_t^{RKKY} = \left(\frac{-2J}{g\mu_t}\right)\vec{m}_t \times \vec{S}_b = -2J\left(\vec{S}_t \times \vec{S}_b\right) = \left|\tau_t^{RKKY}\right|(-\hat{z}), \tag{3}$$

For antiferromagnetic coupling, 'J' is negative, while the projection of $\left(\vec{S}_t \times \vec{S}_b\right)$ is along the –z direction. Therefore, the torque on the top DW will be directed along the –z direction, which will help to flip the top DW into –z direction to expand the 'Down' domain and move along the electron flow direction.

Similarly, the torque on the bottom DW is along +z direction and can be given by

$$\tau_b^{RKKY} = -\vec{m}_b \times \vec{B}_b^{RKKY} = -2J\left(\vec{S}_b \times \vec{S}_t\right) = \left|\tau_b^{RKKY}\right|(\hat{z}), \tag{4}$$

i.e. the bottom DW will flip in +z-direction to expand the 'Up' domain and move along the electron direction. In summary, in both layers the DWs move along the electron flow direction.

From equation 2 & 3, it can be concluded that RKKY antiferromagnetic coupling induced torque greatly depends on the angle between the two DWs. Hence, the perturbation in

the antiferromagnetic coupling due to SHE plays an important role to stimulate the RKKY torques. Therefore, the top and bottom interfaces should be engineered in such a way that SHE torque rotates both the DWs in same directions which further enhances the RKKY torques. In this work the enhanced RKKY torques are achieved by placing Ta and Pt at opposite interfaces. Based on our calculations, effects of capping and seed layers on the DW dynamics in SAF nanowires are summarized in a Table S1.

Fig. S4 (c) shows the M_y-component of the DWs for two different current densities. The M_y-component of the two DWs increases with higher current density due to the SHE. Simulation snapshots of the DW configurations at these two current densities are also added in Fig. S4 (d). At low current density, the antiferromagnetic coupling is more dominant and thus the two DWs are still antiparallel to each other. The non-collinear factor increases with the current density which further increases the contribution from the exchange torque to the DW dynamics.

	DW configuration	Direction of SHE	RKKY torque
**J=0**	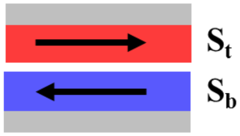	SHE=0	Cross product of two spins is zero. $\tau_{RKKY}=0$
**$J_a>0$**	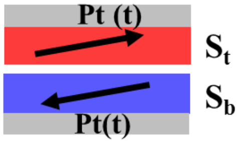	Inversion symmetry. SHE at both the DWs is same in magnitude but opposite in direction.	Cross product of two spins is zero. $\tau_{RKKY}=0$
	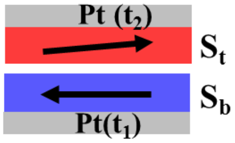	SHE at both the DWs is different in magnitude but opposite in direction. Here $t_1 \neq t_2$.	Cross product of two spins is non-zero. $(\tau_{RKKY})_{(Pt/Pt)}$ **is nonzero [R2]**
	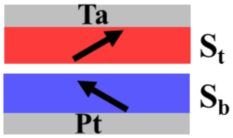	Inversion asymmetry SHE at both the DWs is different in magnitude but same in direction.	Cross product of two spins is non-zero. $(\tau_{RKKY})_{(Pt/Ta)} > (\tau_{RKKY})_{(Pt/Pt)}$

Table S1. Role of spin Hall effect from capping and seed layers to stimulate the RKKY torques.

Supplementary References

R1 Kittel, C. Introduction to solid state physics, 7th edition, *Wiley Singapore*, p.450 (2007).

R2 Yang, S. H., Ryu, K. S. & Parkin, S. Domain-wall velocities of up to 750 m s^{-1} driven by exchange-coupling torque in synthetic antiferromagnets. *Nat. Nano.* **10**, 221 (2015).
